# *Pseudomonas canadensis* sp. nov., a biological control agent isolated from a field plot under long-term mineral fertilization

**DOI:** 10.1099/ijsem.0.001698

**Published:** 2017-05-05

**Authors:** James T. Tambong, Renlin Xu, Eden S. P. Bromfield

**Affiliations:** Ottawa Research and Development Centre, Agriculture and Agri-Food Canada, 960 Carling Avenue, Ottawa, Ontario K1A 0C6, Canada

**Keywords:** *Pseudomonas canadensis* sp. nov., biological control, genome sequencing, ANIm, digital DNA-DNA hybridization (dDDH), polar lipids

## Abstract

The bacterial strain 2-92^T^, isolated from a field plot under long-term (>40 years) mineral fertilization, exhibited *in vitro* antagonistic properties against fungal pathogens. A polyphasic approach was undertaken to verify its taxonomic status. Strain 2-92^T^ was Gram-reaction-negative, aerobic, non-spore-forming, motile by one or more flagella, and oxidase-, catalase- and urease-positive. The optimal growth temperature of strain 2-92^T^ was 30 °C. 16S rRNA gene sequence analysis demonstrated that the strain is related to species of the genus *Pseudomonas*. Phylogenetic analysis of six housekeeping genes (*dnaA*, *gyrB*, *recA*, *recF*, *rpoB* and *rpoD*) revealed that strain 2-92^T^ clustered as a distinct and well separated lineage with *Pseudomonassimiae* as the most closely related species. Polar lipid and fatty acid compositions corroborated the taxonomic position of strain 2-92^T^ in the genus *Pseudomonas*. Phenotypic characteristics from carbon utilization tests could be used to differentiate strain 2-92^T^ from closely related species of the genus *Pseudomonas*. DNA–DNA hybridization values (wet laboratory and genome-based) and average nucleotide identity data confirmed that this strain represents a novel species. On the basis of phenotypic and genotypic characteristics, it is concluded that this strain represents a separate novel species for which the name *Pseudomonas canadensis* sp. nov. is proposed, with type strain 2-92^T^ (=LMG 28499^T^=DOAB 798^T^). The DNA G+C content is 60.30 mol%.

Species of the genus *Pseudomonas* are aerobic, Gram-reaction-negative gammaproteobacteria, ubiquitous in agricultural soils, and are well adapted to grow in the rhizosphere. This genus includes species that are of significant environmental importance such as plant growth promoters, xenobiotic degraders and biocontrol agents [1–3]. The fluorescent pseudomonads are uniquely capable of synthesizing many metabolites that play a role in maintaining soil health leading to bioprotection of crops against pathogens [4, 5].

In 2009, we started a prospective study focused on cultivable members of the genus *Pseudomonas* in a soil ecosystem under long-term (>40 years) applications of inorganic fertilizer (NPK). Soil samples were collected from a corn-alfalfa rotation plot located at Woodslee, Ontario, Canada (42.22 N 82.73 W). Triplicates of bulk soil (10 g each) were suspended in 90 ml 0.85 % NaCl and vortexed vigorously. The soil suspensions were serially diluted in 0.85 % NaCl, plated on King’s B (KB) agar (Sigma-Aldrich) and incubated at 28 °C for 48 h to isolate fluorescent pseudomonads. Colonies were screened for fluorescence under UV light. Single colonies were obtained after repeated streaking and plated on Pseudomonas-F agar medium (BD Difco). A total of 148 bacterial isolates were obtained of which 99 % fluoresced under UV light. All isolates were evaluated for *in vitro* antagonistic activities against the following fungal pathogens: *Fusarium graminearum*, *Rhizoctonia solani* and *Gaeumannomyces graminis*. Twelve of the isolates were able to inhibit the mycelial growth of at least two of the fungal pathogens. All 12 biological control isolates were identified as members of the genus *Pseudomonas* on the basis of phenotypic features and 16S rRNA sequence analyses. Of the three strains (2–92, 2–36 and 2–114) that potently inhibited the growth of *R. solani* and *G. graminis,* two were assigned to known species of the genus *Pseudomonas*: *Pseudomonas simiae* 2–36 and *Pseudomonas extremorientalis* 2–114. Strain 2–92^T^ did not match any known species of the genus *Pseudomonas*.

In the present study, the taxonomic status of strain 2–92^T^ was investigated using electron microscopy, phenotypic tests, chemotaxonomic traits, analyses of the 16S rRNA gene and six house-keeping genes (*dnaA*, *gyrB*, *recA*, *recF*, *rpoB* and *rpoD*), DNA–DNA hybridization and DNA G+C content determination as well as analysis of draft genome sequences. Based on this polyphasic characterization, a novel species, *Pseudomonas canadensis* sp. nov. is proposed.

Cells of strain 2-92^T^ were purified on Pseudomonas-F agar medium (BD Difco), and cell suspensions in Luria–Bertani (LB; BD Difco) broth supplemented with 30 % (v/v) glycerol were maintained at −80 °C. Cells were routinely grown overnight in liquid LB with shaking or on LB agar medium and incubated at 28 °C. Strain 2-92^T^ was Gram-reaction-negative based on the 3 % KOH assay [6] and oxidase-positive based on API 20 NE strips (BioMérieux). It was catalase-positive based on 3 % (v/v) hydrogen peroxide solution. Cell growth was tested at different temperatures (5–40 °C, in steps of 1 °C below 5 °C then at intervals of 5 °C), and salt tolerance (NaCl) was determined in the range 0–6 % (w/v) as described by González *et al.* [7]. Strain 2-92^T^ grew at 4 °C, showed optimal growth at 30 °C and did not grow at 40 °C. This strain was tolerant to different NaCl concentrations up to 4 % and was non-spore-forming based on the Schaeffer and Fulton method [8]. Cell morphology was investigated using scanning (SEM) and transmission (TEM) electron microscopy. Bacteria were cultured in LB broth overnight at 28 °C, processed as described by Greco-Stewart *et al.* [9] and imaged using a Philips XL-30 ESEM scanning electron microscope (data not shown). TEM was performed as previously reported by Hayat and Miller using 1 % phosphotungstic acid (pH 7.0) [10], and images were captured with a Hitachi H7000 microscope (Fig. 1). Cells were rod-shaped with sizes in the range of 0.5–0.6 µm wide and 2.1–2.6 µm long, consistent with species of the genus *Pseudomonas* [3], and had one or more polar flagella. Motility was demonstrated using triphenyl tetrazolium [11] in semisolid medium (per litre: 3.0 g beef extract, 10.0 g pancreatic digest of casein, 5.0 g NaCl, 4.0 g agar). Fluorescent pigment was produced on KB medium [12]. After 48 h of incubation at 28 °C on KB, the colonies of 2-92^T^ were white–yellowish, circular (mean 4 mm in diameter) and convex with regular margins.

**Fig. 1.**
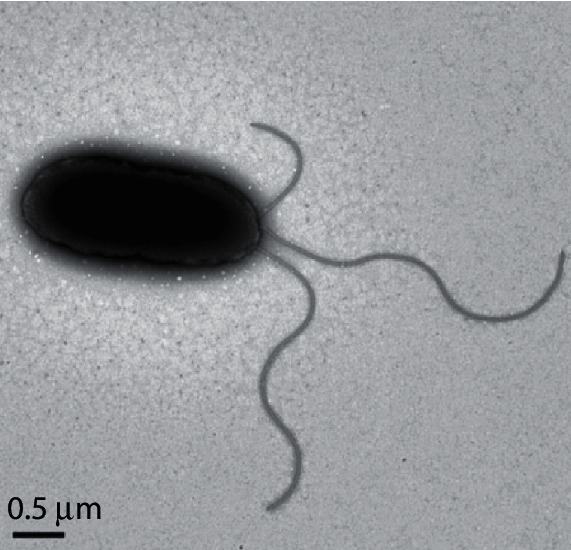
Transmission electron micrograph of a cell of strain 2-92^T^ from an overnight culture showing multiple flagella. Bar, 0.5 µm.

The cellular fatty acid composition of 2-92^T^ was determined by Keystone labs (Alberta Canada) using an Agilent Technologies 6890 N gas chromatograph. Strain 2-92^T^ and closely related type strains were grown, in parallel, on trypticase soy broth (TSB) agar (TSBA; 30 g l^−1^ TSB, 15 g l^−1^ agar; BD Biosciences) at 28.0 °C for 24 h, and fatty acid extraction and analyses were performed according to the recommendations of the Microbial Identification (MIDI) system. The profiles were generated and identified using the Microbial Identification System, Sherlock TSBA60 Library version 6.0 (MIDI). Profiles of strain 2-92^T^ were compared with those of closely related species of the *Pseudomonas fluorescens* subgroup generated under the same standardized conditions (Table S1, available in the online Supplementary Material). The major cellular fatty acid peaks (Table S1) of strain 2-92^T^ were C_16 : 0_ (31.3 %), C_16 : 1_ω6*c*/C_16__ : 1_ω7*c* (19.9 %; summed feature 3), C_18 : 1_ω7*c*/C_18__ : 1_ω6*c* (14.4 %; summed feature 8), C_17 : 0_ cyclo (9.2 %), C_12 : 0_ 2-OH (7.7 %), C_12 : 0_ 3-OH (6.1 %) and C_10 : 0_ 3-OH (3.2 %). The presence of C_10 : 0_ 3-OH and C_12 : 0_ 3-OH fatty acids in the profile of strain 2-92^T^ is consistent with the classification as a *sensu stricto* pseudomonad [13]. In addition, the polar lipids of strain 2-92^T^ were determined by the Identification Service of the DSMZ (Braunschweig, Germany). The assay identified major amounts of phosphatidylethanolamine, diphosphatidylglycerol and phosphatidylglycerol (Fig. S1), which is consistent with species of the genus *Pseudomonas* [14]. Minor amounts of phosphatidylcholine, unidentified phospholipid and unidentified lipid were also detected (Fig. S1).

API 20 NE strips (BioMérieux) and carbon utilization tests based on Biolog PM1 and PM2A MicroPlates were performed according to the instructions of the manufacturers (Table 1). In API 20 NE assays performed in parallel with the 7 closely related type strains, strain 2-92^T^ assimilated potassium gluconate, capric acid, adipic acid and trisodium citrate, and was positive for arginine dihydrolase, urease, gelatinase and oxidase but negative for indole production, β-glucosidases and β-galactosidase. Using Biolog assays, in a parallel study with closely related strains, strain 2-92^T^ could be differentiated from *P. simiae* CCUG 50988^T^ (phylogenetically closest species) by its ability to assimilate sucrose, trehalose, melibiose, l-rhamnose, l-phenylalanine, d-xylitol, *N*-acetyl-d-glucosamine, itaconic acid and putrescine as well as its inability to reduce nitrates (Table 1). Also, strain 2-92^T^ could be differentiated from the type strains *Pseudomonasazotoformans* LMG 21611^T^, *P. costantinii* LMG 22119^T^, *P. extremorientalis* LMG 19695^T^, *P. poae* LMG 21465^T^, *P. salomonii* LMG 22120^T^, *P. simiae* CCUG 50988^T^ and *P. trivialis* LMG 21464^T^ [15–19] by using several phenotypic characteristics (Table 1).

**Table 1.** Phenotypic characteristics that differentiate *P. canadensis* sp. nov. 2–92^T^ from closely related species of the genus *Pseudomonas* Strains: 1, *P. canadensis* sp. nov. 2-92^T^ (=LMG 28499^T^); 2, *P. simiae* CCUG 50988^T^; 3, *P. extremorientalis* KMM 3447^T^; 4, *P. costantinii* CFBP 5705^T^; 5, *P. salomonii* LMG 22120^T^; 6, *P. poae* DSM 14936^T^; 7, *P. trivialis* DSM 14937^T^; 8, *P. azotoformans* LMG 21611^T^. *P. canadensis* sp. nov. 2–92^T^ can be distinguished from the reference taxa by its ability to utilize melibiose and l-rhamnose, except for *P. trivialis* that showed a variable reaction for l-rhamnose. *P. canadensis* sp. nov. can be differentiated from *P. simiae* by its ability to assimilate sucrose, melibiose, l-rhamnose, l-phenylalanine, *N*-acetyl-d-glucosamine, itaconic acid and putrescine. +, Positive reaction; −, negative reaction; w, weakly positive reaction; v, variable reaction.

	1	2	3	4	5	6	7	8
Nitrate reduction	−	+	−	−	−	−	−	nd
Aesculin hydrolysis	−	−	nd	−	−	+	+	−
Gelatinase	+	+	−	+	+	v	v	−
Assimilation of:								
Sucrose	+	−	+	v	+	+	−	−
Maltose	−	−	+	−	+	−	−	−
Trehalose	+	−	+	+	+	+	+	+
* α*-d-Glucose	+	+	−	+	+	v	+	−
d-Galactose	+	+	+	+	+	+	+	+
Lactose	−	−	−	−	−	−	−	−
Melibiose	+	−	−	−	−	−	−	−
l-Rhamnose	+	−	−	−	−	−	v	−
d-Mannitol	+	+	+	+	+	+	+	−
l-Phenylalanine	+	−	−	+	−	−	−	+
* N*-Acetyl-d-glucosamine	+	−	+	+	−	+	−	+
i-Erythritol	+	+	−	+	+	−	−	+
d-Xylitol	+	−	+	+	+	−	−	+
Adonitol	+	+	−	+	+	−	−	+
*myo*-Inositol	+	+	−	+	+	+	+	+
Itaconic acid	+	−	+	+	+	v	v	+
Putrescine	+	−	−	+	+	+	v	+
l-Ornithine	+	+	−	+	+	−	−	+

Genomic DNAs were extracted using a Wizard SV Genomic DNA Purification kit (Promega), and the purity of each sample was determined by agarose gel electrophoresis. The DNA concentrations were determined fluorimetrically using a FLUOstar OPTIMA micro-plate reader (BMG-LABTECH) with picogreen chemistry (Invitrogen). PCR amplifications of almost full-length (1466 bp) 16S rRNA genes were performed as described previously [20] using primer pairs 16F27 (5′-AGAGTTTGATCMTGGCTCAG-3′) and 16R1492 (5′-TAC GGYTACCTTGTTACGACTT-3′) [21]. Sequencing (ABi 3300xl analyzer; Applied Biosystems) of the DNA fragments was performed in-house as reported previously [22] using BigDye terminator chemistry. All the other 16S rRNA gene sequences (53) of reference species of the genus *Pseudomonas* were retrieved from GenBank. Sequences of strain 2-92^T^ were compared with other species by pairwise distance calculations; multiple sequence alignments and phylogenetic analysis of the 16S rRNA gene sequences were performed using muscle [23] and mega5.0 [24], respectively. Phylogenetic analysis was performed on almost-complete 16S rRNA gene sequences using maximum-likelihood (ML) with a general time reversible best-fit substitution model as implemented in jModelTest version 2 [25]. The topological robustness of the trees was evaluated by bootstrap analysis based on 1000 replicates. The 16S rRNA gene tree confirmed that strain 2-92^T^ was unique and a member of the genus *Pseudomonas* (Fig. 2). Pairwise sequence similarity values were between 98.8 and 99.5 % with several type strains of the *P. fluorescens* group [26], for example *P. simiae* CCUG 50988^T^, *P. salomonii* LMG 22120, *P. extremorientalis* KMM 3447^T^ and *P. azotoformans* LMG 21611^T^ (Fig. 2). Phylogenies reconstructed using neighbour-joining and minimum-evolution algorithms showed similar topologies (Fig. S2).

**Fig. 2.**
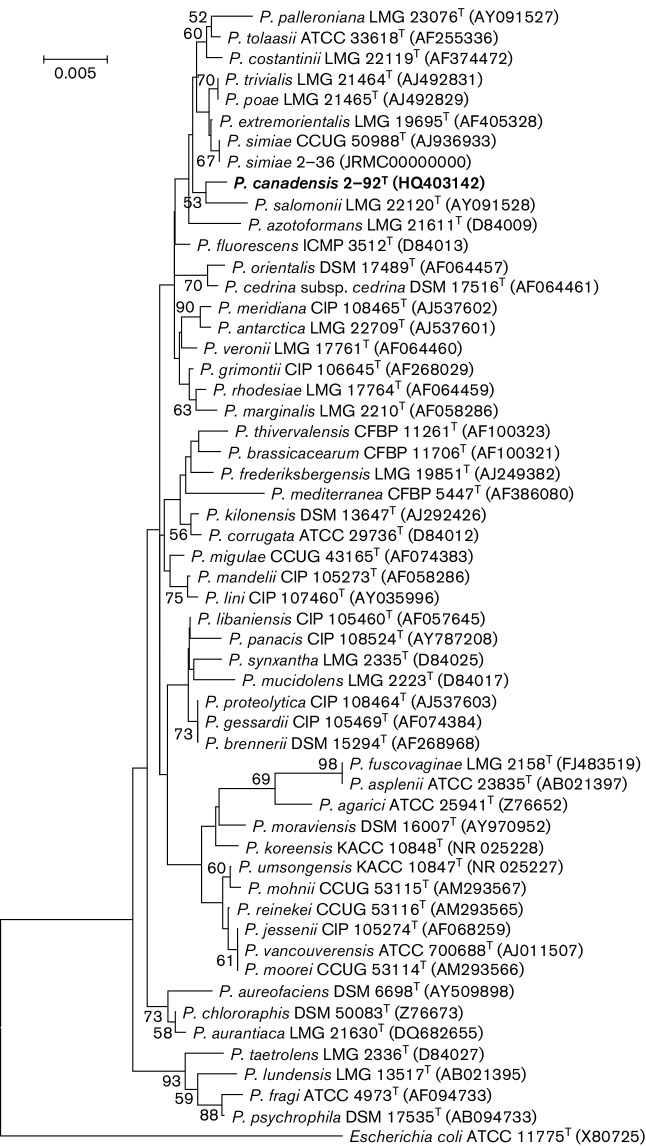
ML tree based on 16S rRNA (1410 bp) gene sequences, showing the taxonomic position of *P.canadensis* sp. nov. 2-92^T^ within the genus *Pseudomonas*. The ML tree was reconstructed using the general time reversible substitution model based on 1000 bootstrap replicates. Bootstrap values >50 % are indicated at nodes. Bar, sequence divergence.

Consistent with previous reports [7, 26] 16S rRNA gene sequences showed low resolution at the intrageneric level. Housekeeping genes such as *rpoD*, *gyrB* and *rpoB* have been used routinely to refine interspecfic phylogenetic positions of species of the genus *Pseudomonas* [7, 26, 27]. Partial gene fragments of *dnaA*, *gyrB*, *recA*, *recF*, *rpoB* and *rpoD* were retrieved from *de novo*-assembled draft genomes obtained in this study from seven closely related species of the genus *Pseudomonas*. The whole-genome sequences were determined by paired-end sequencing using an Illumina MiSeq instrument with TrueSeq V3 chemistry (Génome-Québec, Montreal, Canada). *De novo* assembly was performed using ABySS version 1.5.2 [28] at different k-mer values (75–113) as previously reported [29]. The GenBank accession numbers of the draft genomes used in this study are AYTD00000000 [30], MDDQ00000000, MDDR00000000, MDGK00000000, MDFK00000000, MDFI00000000, MDFH00000000 and MDFJ00000000 for strain 2-92^T^, *P. azotoformans* LMG 21611^T^, *P. costantinii* LMG 22119^T^, *P. extremorientalis* LMG 19695^T^, *P. poae* LMG 21464^T^, *P. salomonii* LMG 22120^T^, *P. simiae* CCUG 50988^T^ and *P. trivialis* 21 464^T^, respectively. A ML tree of *dnaA*-*gyrB*-*recA*-*recF*-*rpoB*-*rpoD* concatenated genes (~6 kb) was inferred using the general time reversible substitution model and implemented in mega5 (Fig. 3). In the *dnaA*-*gyrB*-*recA*-*recF*-*rpoD*-*rp*o*D* concatenated tree, strain 2-92^T^ was placed in a single lineage supported by a high bootstrap value (99 %) with *P. simiae* CCUG 50988^T^ as its closest neighbour. This is consistent with the pairwise similarity values of concatenated genes computed with Geneious (www.geneious.com, [31]; Table S2). Phylogenies reconstructed using the neighbour-joining and minimum-evolution algorithms showed similar topologies (data not shown).

**Fig. 3.**
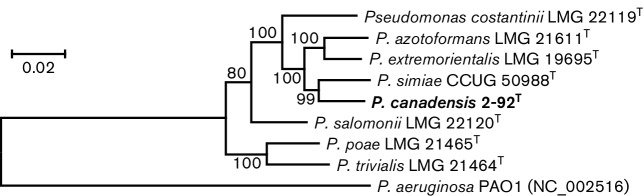
*dnaA*-*gyrB*-*recA*-*recF*-*rpoB*-*rpoD* (~6 kb) ML phylogeny showing the taxonomic position of *P.canadensis* sp. nov. 2-92^T^ relative to closely related species of the genus *Pseudomonas*. The ML tree was reconstructed using the general time reversible substitution model based on 1000 bootstrap replicates. Bootstrap values are indicated at branch points. Bar, sequence divergence.

DNA–DNA hybridization (DDH) studies were employed as previously described by Ramisse *et al.* [32] between strain 2-92^T^ and seven closely related species of the genus *Pseudomonas* (Table 2). Hybridizations were performed in triplicates with reciprocal reactions. DDH values of 49.0, 35.6 and 46.7 % between strain 2-92^T^ and *P. simiae* CCUG 50988^T^, *P. extremorientalis* LMG 19695^T^ and *P. salomonii* LMG 22120^T^, respectively (Table 2), were clearly below the threshold level (<70 %) for species definition [33]. Also, genome-sequence-based digital DDH (dDDH; [34]) and MUMmer-based average nucleotide identity (ANIm; [35]), which have been proposed to replace wet-lab DDH [34, 36], were employed to confirm the taxonomic position of strain 2-92^T^. The dDDH values were calculated using the genome-to-genome distance calculator (GGDC) version 2.1 (http://ggdc.dsmz.de; [34]). ANIm similarity values were computed as described by Kurtz *et al.* [35] and implemented in JSpecies [37]. The dDDH values between strain 2-92^T^ and the seven closely related species of the genus *Pseudomonas* were all below the threshold of 70 % for species delineation (Table 2) as recommended by Meier-Kolthoff *et al*. [34]. For example, strain 2-92^T^ had dDDH values of 45.60 and 33.45 % with *P. simiae* CCUG 50988^T^ and *P. salomonii* LMG 22120^T^, respectively. Similarly, the ANIm values (88.1–90.7 %)  between strain 2-92^T^ and *P. simiae* CCUG 50988^T^ (92.0 %) or the other closely related species were below the threshold level (<95 %) for species definition as reported by Richter and Rosselló-Móra [37] (Table 2). The DNA G+C content of strain 2-92^T^ was 60.3 mol% using the HPLC method [38], which is well within the range reported for species of the genus *Pseudomonas* [3]. The DNA G+C content was confirmed by whole-genome sequencing and analysis of strain 2-92^T^ (AYTD00000000; 6.4 Mb genome size) [30].

**Table 2.** Wet-lab and genome-based DDH values between *P.canadensis* sp. nov. and the type strains of closely related species of the genus *Pseudomonas* based on 16S rRNA gene phylogeny and multi-locus sequence analysis Reciprocal values are given in parentheses. Genome-to-genome digital DDH (dDDH) values were computed using the program ggdc 2.1 [34]. MUMmer-based average nucleotide identity (ANIm) values were computed using the program JSpecies [37].

	Wet-lab DDH (%)	dDDH (%)	ANIm (%)
Type strain	2-92^T^	2-92^T^	2-92^T^
*P. canadensis* 2-92^T^	100	100	100
*P. simiae* CCUG 50988^T^	49.0±2.1 (48.4)	45.60 (45.50)	92.02 (92.09)
*P. trivialis* LMG 21464^T^	45.1±2.8 (48.5)	45.40 (45.07)	88.2 (87.98)
*P. poae* LMG 21465^T^	38.5±3.8 (42.0)	32.15 (34.01)	88.1 (87.9)
*P. salomonii* LMG 22120^T^	46.7±3.2 (47.2)	33.45 (34.01)	88.7 (88.54)
*P. costantinii* LMG 22119^T^	48.3±3.1 (50.1)	33.70 (33.10)	88.8 (88.4)
*P. azotoformans* LMG 21611^T^	45.0±4.0 (51.0)	40.20 (40.09)	90.68 (90.74)
*P. extremorientalis* LMG 19695^T^	35.6±4.2 (40.0)	40.40 (40.4)	90.74 (90.68)

Based on the data from genotypic and phenotypic analyses presented in this study, we propose that the novel strain represents a novel species, named *Pseudomonas canadensis* sp. nov.

## Description of *Pseudomonas canadensis* sp. nov.

*Pseudomonas canadensis* (ca.nad.en′sis N.L. fem. adj. *canadensis* from or originating from Canada, the country where strain 2-92^T^ was isolated).

Cells are aerobic, Gram-reaction-negative, non-spore-forming rods (approx. 0.5 µm wide and 2.3 µm long), motile with one or multiple polar flagella. After 48 h on KB, colonies are white-yellowish and circular (average 4 mm in diameter), convex with regular margins and produce fluorescent pigments. Tolerant to different NaCl concentrations up to 4 %, and grows at 4 °C with optimal growth at 30 °C and no growth at 40 °C. Based on Biolog PM1 and PM2A Microplate assays, utilizes *N-*acetyl-d-glucosamine, succinic acid, l-aspartic acid, l-proline, d-alanine, d-mannose, d-gluconic acid, l-lactic acid, d-mannitol, l-glutamic acid, dl-malic acid, Tween 20, d-fructose, acetic acid, α-d-glucose, l-asparagine, α-ketoglutaric acid, l-glutamine, adenosine, citric acid, fumaric acid, bromosuccinic acid, propionic acid, inosine, l-serine, l-alanine, l-alanyl-glycine, monomethylsuccinate, methylpyruvate, l-malic acid, pyruvic acid, γ-aminobutyric acid, caproic acid, d-glucosamine, β-hydroxybutyric acid, malonic acid, quinic acid, succinamic acid, l-arginine, hydroxy-l-proline, l- isoleucine, l-leucine, l-pyroglutamic acid, l-valine, d,l-carnitine, glycerol, l-arabinose, d-saccharic acid, d-galactose, d-galactonic acid γ-lactone, mucic acid, d-glucosaminic acid and d-arabitol but not lactose, maltose, d-fructose 6-phosphate, α-hydroxyglutaric acid γ-Lactone, glycyl-l-proline, phenylethylamine, 1,2-propanediol, d-glucose 6-phosphate, sebacic acid, Tween 40, Tween 80 or formic acid. Using API 20 NE assays, assimilates potassium gluconate, capric acid, adipic acid and trisodium citrate. Positive for arginine dihydrolase, urease, gelatinase and oxidase but negative for indole production, β-glucosidases and β-galactosidases. The most abundant fatty acids are C_16 : 0_, C_16 : 1_ω7*c* and/or C_16 : 1_ω6c (summed feature 3) and C_18 : 1_ω7*c* and/or C_18 : 1_ω7*c* (summed feature 8).

The type strain is 2-92^T^ (=DOAB 798^T^=LMG 28499^T^), isolated from a soil sample from Woodslee, Ontario, Canada. The DNA G+C content of strain 2-92^T^ is 60.3 mol%.

## Supplementary Data

118 Supplementary File 1
