# Supplementary material for: Pseudomonas canadensis sp. nov., a biological control agent isolated from a field plot under long-term mineral fertilization
Source: Int J Syst Evol Microbiol. 2017 May 5;67(4):889–95. doi: 10.1099/ijsem.0.001698 (PMC5817194; doi:10.1099/ijsem.0.001698)
Supplement: Supplementary File 1 [file ijsem-67-889-s001.pdf]

Table S1. Cellular fatty acid composition (%) of *Pseudomonas canadensis* sp. nov. and closely-related *Pseudomonas* species.

1. *P. canadensis* sp. nov. 2-92^T; 2. *P. simiae* CCUG 50988^T; 3. *P. azotoformans* LMG 21611^T; 4. *P. extremorietalis* LMG 19695^T; 5. *P. poae* LMG 21465^T; 6. *P. trivialis* LMG 21464^T; 7. *P. constantinii* LMG 22119 ^{T}; 8. *P. salomonii* LMG 22120^T. Extraction of fatty acids and analysis were performed Keystone Laboratory, (Alberta, Canada) using MIDI System. -, not detected or trace amounts (<1%). †summed feature 8; # summed feature 3.

Fatty acid	1	2	3	4	5	6	7	8
$C_{10:0}$ 3-OH	3.2	3.0	3.2	-	3.0	2.7	2.5	3.1
$C_{12:0}$	2.2	2.1	3.9	1.4	2.9	2.9	2.8	2.7
$C_{12:0}$ 2-OH	7.7	4.6	2.8	-	4.1	4.1	4.4	4.3
$C_{12:0}$ 3-OH	6.1	4.0	3.5	-	3.9	3.9	4.0	4.1
$C_{16:0}$	31.3	35.1	25.6	37.0	31.9	30.5	31.8	30.8
$C_{17:0}$ cyclo	9.2	3.8	-	31.3	1.2	0.6	6.3	2.5
$C_{18:1}$ ω9c	1.7	-	-	-	-	-	-	-
$C_{18:0}$	2.6	2.6	2.5	1.2	0.7	0.8	0.8	0.9
$C_{18:1}$ ω7c/C18:1 ω6c†	14.4	12.8	22.7	14.0	17.3	18.8	15.7	31.3
$C_{16:1}$ ω7c/C16:1 ω6c#	19.9	32.3	33.9	8.7	34.3	34.7	30.1	19.2

Table S2. Pairwise sequence similarity values of *dna*A-*gyr*B-*rec*A-*rec*F-*rpo*B-*rpo*D concatenated gene fragments between strain 2-92^T and closely related type strains of *Pseudomonas* spp.

Type strains	1	2	3	4	5	6	7	8
1. *P. canadensis* 2-92^T								
2. *P. simiae* CCUG 50988^T	95.39							
3. *P. trivialis* LMG 21464^T	94.39	94.35						
4. *P. poae* LMG 21465^T	94.67	94.64	96.58					
5. *P. salomonii* LMG 22120^T	93.38	93.66	93.42	93.53				
6. *P. costantinii* LMG 22119^T	93.01	92.94	93.11	93.56	93.03			
7. *P. azotoformans* LMG 21611^T	91.18	91.58	91.45	91.65	92.15	91		
8. *P. extremorientalis* LMG 19695^T	90.95	91.35	91.45	91.48	92.07	90.57	94.89	

Fig. S1. A two-dimensional thin layer chromatograph of polar lipids of strain *Pseudomonas canadensis* 2-92^{T} : DPG, diphosphatidylglycerol; L, lipid; PE, Phosphatidylethanolamine; PG, phosphatidylglycerol; PL, phospholipid; PC, Phosphatidylcholine.

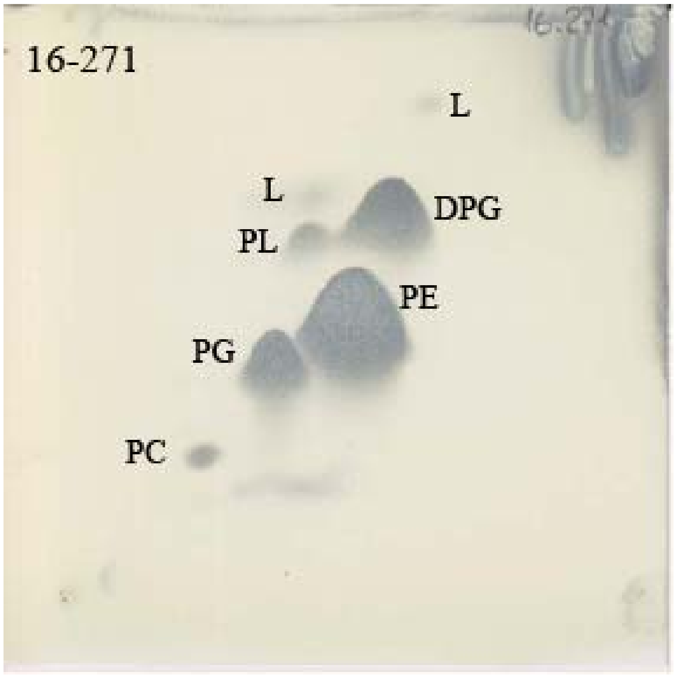

Fig. S2. Neighbor-joining (a) and Minimium evolution (b) trees based on 16S rRNA (1410 bp) gene sequences showing similar taxonomic position of *Pseudomonas canadensis* 2-92^T within the genus *Pseudomonas*. Bootstrap values >50% are indicated at branch points. The bars indicate sequence divergence.

a)

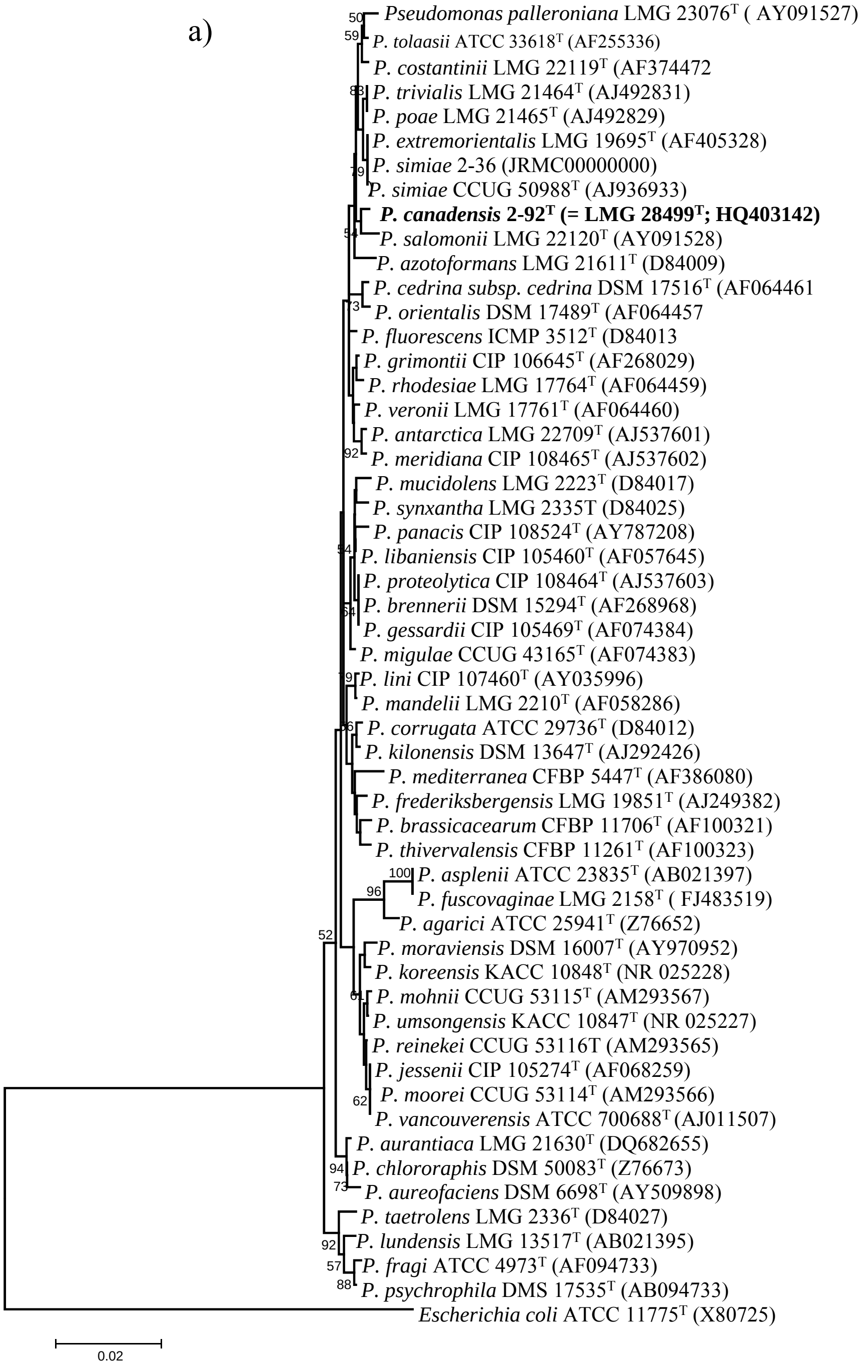

Fig. S2

b)

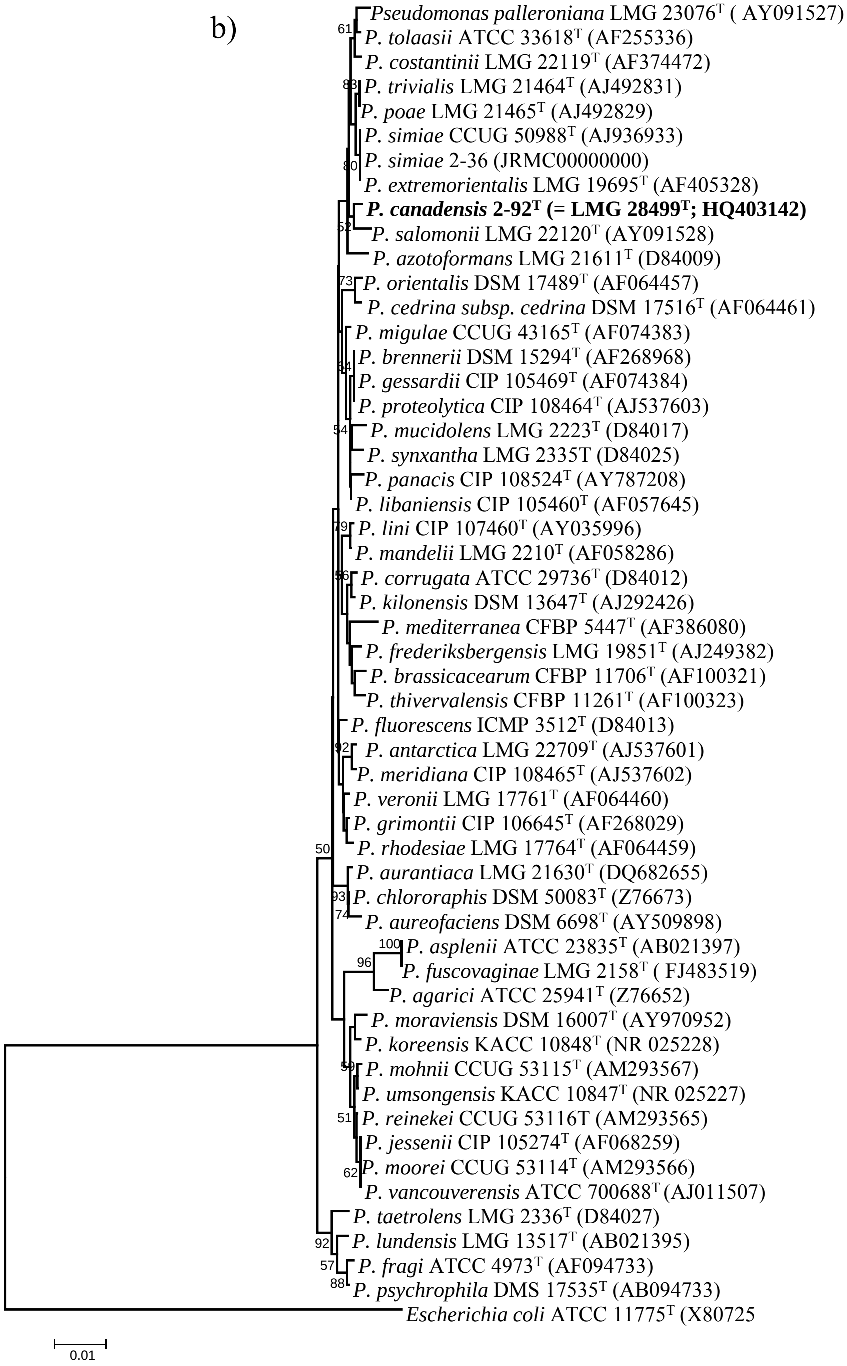
Pseudomonas palleroniana LMG 23076^{T} (AY091527)
P. tolaasii ATCC 33618^{T} (AF255336)
P. costantinii LMG 22119^{T} (AF374472)
P. trivialis LMG 21464^{T} (AJ492831)
P. poae LMG 21465^{T} (AJ492829)
P. simiae CCUG 50988^{T} (AJ936933)
P. simiae 2-36 (JRMC00000000)
P. extremorientalis LMG 19695^{T} (AF405328)
***P. canadensis* 2-92^{T} (= LMG 28499^{T}; HQ403142)**
P. salomonii LMG 22120^{T} (AY091528)
P. azotoformans LMG 21611^{T} (D84009)
P. orientalis DSM 17489^{T} (AF064457)
P. cedrina subsp. cedrina DSM 17516^{T} (AF064461)
P. migulae CCUG 43165^{T} (AF074383)
P. brennerii DSM 15294^{T} (AF268968)
P. gessardii CIP 105469^{T} (AF074384)
P. proteolytica CIP 108464^{T} (AJ537603)
P. mucidolens LMG 2223^{T} (D84017)
P. synxantha LMG 2335T (D84025)
P. panacis CIP 108524^{T} (AY787208)
P. libaniensis CIP 105460^{T} (AF057645)
P. lini CIP 107460^{T} (AY035996)
P. mandelii LMG 2210^{T} (AF058286)
P. corrugata ATCC 29736^{T} (D84012)
P. kilonensis DSM 13647^{T} (AJ292426)
P. mediterranea CFBP 5447^{T} (AF386080)
P. frederiksbergensis LMG 19851^{T} (AJ249382)
P. brassicacearum CFBP 11706^{T} (AF100321)
P. thivervalensis CFBP 11261^{T} (AF100323)
P. fluorescens ICMP 3512^{T} (D84013)
P. antarctica LMG 22709^{T} (AJ537601)
P. meridiana CIP 108465^{T} (AJ537602)
P. veronii LMG 17761^{T} (AF064460)
P. grimontii CIP 106645^{T} (AF268029)
P. rhodesiae LMG 17764^{T} (AF064459)
P. aurantiaca LMG 21630^{T} (DQ682655)
P. chlororaphis DSM 50083^{T} (Z76673)
P. aureofaciens DSM 6698^{T} (AY509898)
P. asplenii ATCC 23835^{T} (AB021397)
P. fuscovaginae LMG 2158^{T} (FJ483519)
P. agarici ATCC 25941^{T} (Z76652)
P. moraviensis DSM 16007^{T} (AY970952)
P. koreensis KACC 10848^{T} (NR 025228)
P. mohnii CCUG 53115^{T} (AM293567)
P. umsongensis KACC 10847^{T} (NR 025227)
P. reinekei CCUG 53116T (AM293565)
P. jessenii CIP 105274^{T} (AF068259)
P. moorei CCUG 53114^{T} (AM293566)
P. vancouverensis ATCC 700688^{T} (AJ011507)
P. taetrolens LMG 2336^{T} (D84027)
P. lundensis LMG 13517^{T} (AB021395)
P. fragi ATCC 4973^{T} (AF094733)
P. psychrophila DMS 17535^{T} (AB094733)
Escherichia coli ATCC 11775^{T} (X80725
0.01
